# Improved Extraction of the Neurotoxin of *Stenocarpella maydis* Using an Artificial Rumen

**DOI:** 10.1155/vmi/4160516

**Published:** 2025-10-17

**Authors:** Leendert Dekker Snyman

**Affiliations:** Toxicology and EVM, ARC-Onderstepoort Veterinary Institute, Private Bag X05, Onderstepoort, Pretoria 0110, South Africa

**Keywords:** diplodiosis, extraction, guinea pig, neurotoxin, sheep, *Stenocarpella maydis*

## Abstract

Due to the low yield of diplonine obtained when isolated from *Stenocarpella maydis* (Berk.) Sutton (formerly *Diplodia maydis* (Berk.) Sacc., the present research was aimed to find a more efficient method of preparing diplonine to confirm its neurological effect on the target animal. This study demonstrated that using an artificial rumen for extraction instead of methanol may significantly improve the extraction of the neurotoxin. With this method, only 1/10^th^ of an *S. maydis* culture was required to induce neurological disorders in guinea pigs than was needed to induce the neurological disorders with a methanol extract from the same culture, indicating a possible superiority of an artificial rumen extract compared to extraction with methanol. A comparable dosage of the artificial rumen extract also induced neurological disorders resembling diplodiosis in sheep.

## 1. Introduction

*Stenocarpella maydis* (Berk) Sutton, a cob-rot fungus that occurs on maize, causes significant production losses when livestock graze on harvested maize fields infected by the fungus. Intake of the infected plant material causes diplodiosis, a neurotoxicosis characterized by stiff hind legs, incoordination, imbalance, frequent falling, lateral recumbency, and paresis/paralysis [1]. Diplodiosis, first reported in South Africa by Van der Bijl in 1914 [2], has more recently also been reported in Australia [3], Argentina [4], and Brazil [5].

The neurotoxin responsible for diplodiosis in livestock has not been proven yet. Two toxic metabolites isolated from cultures of *S. maydis*, namely, diplodiatoxin and dipmatol, did not induce neurological disorders resembling diplodiosis when administered to the experimental animals [6–8]. A third metabolite isolated from *S. maydis*, designated diplonine, induced neurological disorders in guinea pigs, reminiscent of diplodiosis in livestock [9], but it has not yet been tested on the target animal. To achieve this, a substantial quantity of diplonine must be prepared.

The yield of diplonine obtained during its isolation, however, was very low [9], making it laborious and very expensive to prepare for this purpose. The method used involves a crude extraction with methanol followed by repeated fractionation on silica gel, followed by final purification on RP 18 as noted in the paper [9]. This procedure results in significant losses of diplonine.

A subsequent study showed that the crude extraction of the neurotoxin could be significantly improved by using water as the extraction medium instead of methanol [10]. The present study attempted to further improve the crude extraction of diplonine by mimicking the natural extraction process in the rumen. This was performed using an artificial rumen. Extraction of the neurotoxin by this method was compared to extraction with methanol. Evaluation was based on the neurological disorders elicited in sheep and guinea pigs dosed with various dosages of the extracts.

## 2. Materials

A Beckman Coulter Inc. centrifuge was used to centrifuge the artificial rumen extract (ARE), while a Buchi R-100 rotary evaporator was used for evaporating the solvents.

The chemicals and methanol used for extraction were analytical grade Merck products.

*Stenocarpella maydis* cultures were remnants obtained from a previous study [11].

Merino wethers weighing approximately 20 kg and healthy female guinea pigs, approximately 4 weeks old and weighing around 180 g, were used in these trials. The animals were kept in separate housing from male guinea pigs to prevent reproduction. All procedures involving animals were conducted in accordance with the South African National Standard (*The Care and Use of Animals for Scientific Purposes* [SANS 10386:200X]). Animal trials were approved by the Animal Ethics Committee of the Agricultural Research Council, Onderstepoort Veterinary Institute.

## 3. Methods and Results

The effects of artificial rumen and methanol extracts of *S. maydis* on the neurological signs in sheep and guinea pigs were investigated. The extracts were administered to the animals via a stomach tube after being dried and suspended in water.

### 3.1. ARE

Artificial rumen extracts were prepared from *S*. *maydis* cultures following the method described for forages by Tilley and Terry [12] and adapted by Engels and Van der Merwe [13]. Six hundred grams of the *S. maydis* culture were incubated anaerobically for 24 h with 6 L of a medium consisting of 4.8 L of artificial saliva [14] mixed with 1.2 L of filtered rumen fluid. The rumen fluid was withdrawn with suction using a stomach tube from a sheep that was fed on lucerne hay.

### 3.2. Neurological Signs

The neurological signs that developed in sheep and guinea pigs after being dosed with artificial rumen and methanol extracts from *S. maydis* cultures, along with the corresponding ratings, are presented in Table 1.

### 3.3. Neurological Signs in Sheep

Three lambs, identified as Sheep 1, 2, and 3, were dosed with AREs prepared from Cultures 1, 2, and 3, obtained as remnants from a previous study [11]. Culture 1 was the same material as Culture MRC 2829 used in the previous study, while the culture designations for 2 and 3 were unknown. Sheep 1 received the ARE of Culture 1 at a rate of 18 g culture equivalent/kg BW, Sheep 2 received the ARE of Culture 2 at a rate of 23 g culture equivalent/kg BW, and Sheep 3 received the ARE of Culture 3 at a rate of 22 g culture equivalent/kg BW.

**Figure 1:**
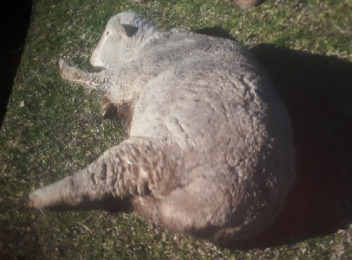
Sheep 3 lying in lateral recumbency 40 h after being dosed with an artificial rumen extract of Culture 3.

**Figure 2:**
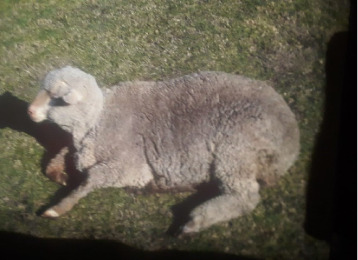
Sheep 3 lying in an upright position 43 h after being dosed with an artificial rumen extract of Culture 3.

The ARE of Culture 1 elicited mild signs of diplodiosis in Sheep 1, displayed by stiff hind legs 22 h after dosing. The ARE of Culture 2 elicited no clinical signs of diplodiosis (unaffected) in Sheep 2, while severe neurological signs of diplodiosis were elicited in Sheep 3 40 h after being dosed with the ARE of Culture 3. The sheep was lying in lateral recumbency, unable to stand up (Figure 1). The sheep then recovered and managed to lie in an upright position 3 hours later (Figure 2), fully recovering 88 h after being dosed (Table 2).

### 3.4. Neurological Signs in Guinea Pigs

#### 3.4.1. Culture 1

**Figure 3:**
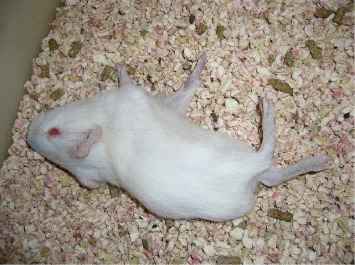
Guinea pig lying in lateral recumbency after being dosed with diplonine [10].

Six guinea pigs were dosed in pairs with the ARE of Culture 1 (MRC 2829) at rates of 19, 38, and 39 g culture equivalent/kg BW. Animals dosed with the 19 and 38 g culture equivalent/kg BW extracts became mildly paretic, showing incoordination 2 h after being dosed. One of the animals that received the higher dose was lying in lateral recumbency at a later stage, while the other one became totally paralyzed and died 4 h after being dosed (Table 2). A guinea pig lying in a state of lateral recumbency after being dosed with diplonine [10] is illustrated in Figure 3. The animals dosed at a rate of 39 g culture equivalent/kg BW also became paralyzed and died 4 and 5 1/2 h after dosing (Table 2).

#### 3.4.2. Culture 3

Two guinea pigs were dosed with AREs of Culture 3. One guinea pig dosed at a rate of 6 g culture equivalent/kg BW became moderately paretic, showing paresis in the hindquarters, while the other guinea pig dosed at a rate of 14 g culture equivalent/kg BW became paralyzed (Table 2). Both guinea pigs fully recovered 48 h after dosing.

## 4. Methanol Extracts

Four hundred grams of the culture were extracted for 16 h with 1 L of methanol on a shaking machine. The extract was centrifuged for 20 min at 1800 × g, the methanol in the supernatant evaporated at 40°C, and the residue was suspended in water.

### 4.1. Neurological Signs in Sheep

Sheep 4 was dosed with a methanol extract of Culture 1 at a rate equivalent to 18 g culture/kg BW. No neurological disorders could be observed afterward, indicating inadequate extraction of the neurotoxin.

### 4.2. Neurological Signs in Guinea Pigs

A methanol extract of Culture 3, prepared as described above, was administered to two guinea pigs at rates of 117 and 124 g culture equivalent/kg BW. The lower dose induced neurological disorders displayed by paresis and mild incoordination, while the animal receiving the higher dose experienced severe incoordination followed by frequent falling. The neurological disorders were observed 6 hours after dosing and disappeared 66 h later (Table 2).

## 5. Discussion

The results of this investigation suggest that extracting an *S. maydis* culture using an artificial rumen may significantly improve the yield of diplonine compared to its extraction with methanol, as previously performed [9]. To induce neurological disorders in guinea pigs, approximately 10 times less *S. maydis* culture was needed when extracting it using an artificial rumen compared to extraction with methanol. In agreement with this, a similar dosage caused neurological signs in a sheep when administered as an ARE but not when given as a methanol extract. The improved extraction can be ascribed to the fact that water is a more effective solvent to extract the neurotoxin than methanol, approximately five times [10]. The superior extraction with the artificial rumen, in addition to its solubility in water, may also be attributed to more favorable conditions such as the higher temperature (39°C) and a possible influence of electrolytes on the solubility of diplonine [15], a substituted β-cyclopropylamino acid [9].

The potency of the ARE compares well with that of the corresponding culture [1], as approximately equal doses of each were required to induce neurological disorders in sheep. This indicates effective extraction of the neurotoxin by the artificial rumen. It should be noted, however, that the culture was administered in two halves, 2 weeks apart, and not in one as performed with the ARE. In addition, dosing the neurotoxin in its extracted form may have further enhanced the potency of the ARE.

Guinea pigs appeared to be at least as sensitive to the neurotoxic effect of the ARE as sheep.

Approximately the same dosage was needed to induce mild neurological disorders in a sheep as in guinea pigs. Higher doses resulted in severe to fatal poisoning of the guinea pigs. In the case of Culture 3, even lower doses of the ARE elicited neurological disorders in guinea pigs compared to sheep. The induction of neurological disorders in guinea pigs excludes the possibility of a pretoxin needing to be changed to its active form in the rumen, suggesting that nonruminants might also be affected by the neurotoxin, including humans.

The neurological disorders induced in the guinea pigs by both artificial rumen and methanol extracts were reminiscent of diplodiosis in livestock. The neurological disorders described, namely, imbalance, frequent falling, paresis in the hindquarters, lateral recumbency, and paralysis, are typical neurological signs of diplodiosis in livestock [1]. The neurological disorders in the sheep dosed with the ARE also resembled diplodiosis in livestock [1]. This was the first time since Mitchell [16] experimentally reproduced diplodiosis in cattle with *Diplodiaceae* (=*S. maydis*) that an extract capable of inducing diplodiosis in a ruminant (sheep) has been made.

To fully benefit from the enhanced extraction of the neurotoxic fraction, subsequent improvement in the purification of diplonine needs to be addressed. The implementation of ion exchange chromatography during the initial steps of fractionation [10] should be considered as a means of doing so.

## 6. Conclusion

The results obtained in this study suggest that the extraction of *S. maydis* using an artificial rumen may significantly enhance the yield of the neurotoxic fraction. With improved purification of the neurotoxic fraction, sufficient quantities of diplonine may be prepared for testing on the target animal.

## Figures and Tables

**Table 1:** Neurological signs observed in experimental animals following administration of *S. maydis* extracts and the corresponding ratings.

Neurological signs	Rating
No signs of poisoning	Unaffected
Mildly paretic with slight incoordination	Mild
Paresis in the hindquarters and signs of imbalance accompanied by frequent falling	Moderate
Animals lying on their side in a state of lateral recumbency	Severe
Animals in a state of paralysis with hind limbs stretched backwards	Extreme
Animals died	Fatal

**Table 2:** Effect of *Stenocarpella maydis* culture extracts and dosing rates on the degree of neurological signs elicited in sheep and guinea pigs.

Animal type	Culture number	Extract dosed	Dosing rate (g culture equivalent per kg BW)	Degree of neurological signs
Sheep 1	1	ARE	18	Mild
Sheep 2	2	ARE	23	Unaffected
Sheep 3	3	ARE	22	Severe
Sheep 4	1	Methanol	18	Unaffected
Guinea pig	1	ARE	19	Mild
Guinea pig	1	ARE	19	Mild
Guinea pig	1	ARE	38	Severe
Guinea pig	1	ARE	38	Fatal
Guinea pig	1	ARE	39	Fatal
Guinea pig	1	ARE	39	Fatal
Guinea pig	3	ARE	6	Moderate
Guinea pig	3	ARE	14	Extreme
Guinea pig	3	Methanol	117	Mild
Guinea pig	3	Methanol	124	Moderate

Abbreviation: ARE, artificial rumen extract.

## Data Availability

The data that support the findings of this investigation are available upon reasonable request.
